# Enteral Feeding/Total Fluid Intake Ratio Is Associated With Risk of Bronchopulmonary Dysplasia in Extremely Preterm Infants

**DOI:** 10.3389/fped.2022.899785

**Published:** 2022-05-31

**Authors:** Bingchun Lin, Xiaoyun Xiong, Xia Lu, Jie Zhao, Zhifeng Huang, Xueyu Chen

**Affiliations:** Department of Neonatology, Shenzhen Maternity and Child Healthcare Hospital, Shandong University, Shenzhen, China

**Keywords:** enteral feeding, nutrition, bronchopulmonary dysplasia, extremely preterm infant, lung development

## Abstract

**Background:**

Nutrition is an essential factor in preventing and managing bronchopulmonary dysplasia (BPD), a multifactorial chronic respiratory disease in premature infants. This study examined the association between nutritional intakes during the first 2 weeks of life and BPD in extremely preterm infants.

**Methods:**

A retrospective single-center cohort study was performed in infants born <28 weeks' gestational age or with a birth weight <1,000 g. Intake of energy and ratio of enteral feeding/ total fluid intake during the first 2 weeks of life and association with outcome of BPD were examined.

**Results:**

134 infants were included in our study, and 43 infants (32.1%) developed BPD. During the first 2 weeks of life, the average of total caloric intake and the ratio of enteral feeding/ total fluid intake were significantly lower in the BPD group (total caloric intake:91.90 vs. 95.72 kcal/kg/d, *p* < 0.05, ratio of enteral feeding/total fluid intake: 0.14 vs. 0.18, *p* < 0.05), while the average of total fluid intake, caloric and protein intake from parenteral nutrition did not differ between the groups. The ratio of enteral feeding/ total fluid intake during the second week were significantly lower in the BPD group (0.21 vs. 0.28, *p* < 0.05), while this ratio during the first week did not differ between the groups. An increase of 10% in the ratio of enteral feeding/ total fluid intake during the second week of life significantly reduced the risk of BPD (OR 0.444, 95% CI: 0.270–0.731).

**Conclusions:**

A higher ratio of enteral feeding/ total fluid intake was associated with a lower risk for BPD. Early and rapidly progressive enteral nutrition should be encouraged in extremely preterm infants in the absence of feeding intolerance.

## Introduction

Bronchopulmonary dysplasia (BPD) is a multifactorial chronic respiratory disease in preterm newborns who underwent mechanical ventilation and oxygen therapy (1). Despite advances in perinatal and neonatal care over the past decades, BPD has remained a significant complication in extremely preterm infants, affecting the prognosis and quality of life. More and more attention has been paid to nutrition, which plays a critical role in preventing and managing BPD (2, 3). In the last decade, optimizing nutrition management in extremely preterm infants has been an effective strategy to improve the quality of neonatal intensive care (4). However, there remain lots of details to explore.

It has been reported that insufficient nutritional intake within the first several days of life may have long-lasting effects on the lung development, maturation and repairment of damage in extremely preterm infants, in whom alveolarization of the lung occurs mostly or entirely after birth (5–7). These studies have reported a lower total energy intake in the first postnatal weeks of life associated with higher risk of BPD. However, some studies have shown that early fluid overload in extremely low birth weight infants is associated with BPD or higher mortality rate (8, 9). A meta-analysis has shown that with restricted water intake, there are trends toward reduced risks of BPD (10). Combining the need for high energy and the limit in fluid intake remains a challenge to clinicians. The balance of energy and fluid intake in the early postnatal days remains debatable due to limited and inconsistent data. This study sought to investigate whether there is an association between early nutrition management and BPD in extremely preterm infants, focusing on caloric intake, enteral feeding, and total fluid intake.

## Methods

### Patients

**Figure 1:**
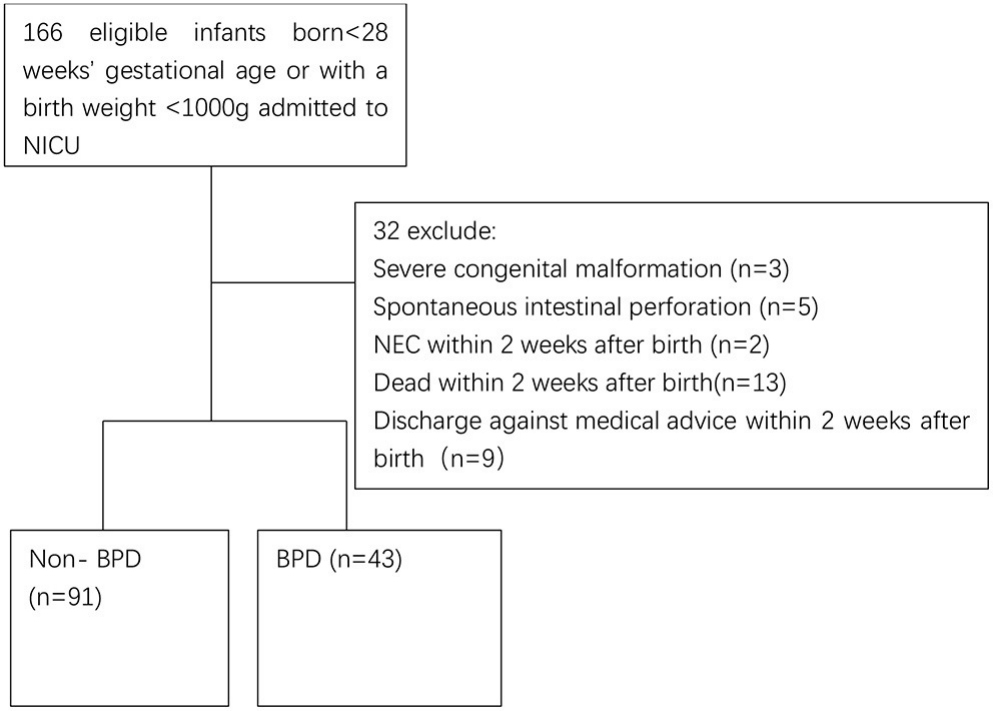
Flowchart of include infants. NEC, necrotizing enterocolitis; BPD, bronchopulmonary dysplasia.

We carried out the present retrospective study at the neonatal intensive care unit (NICU) of Shenzhen Maternal and Child Healthcare Hospital, China, with the approval of the hospital Institutional Medical Ethics Committee (SFYLS [2019] No. 119). The ethics committee waived the informed consent due to the retrospective nature. We reviewed the medical recordings of infants born <28 weeks' gestational age (GA, ELGAN) or with a birth weight <1,000 g (ELBW) born from January 2019 to August 2020. In the 166 recorded extremely preterm infants (Figure 1), 32 were excluded due to the following reasons, severe congenital malformation (*n* = 3), spontaneous intestinal perforation (*n* = 5), necrotizing enterocolitis (NEC) within 2 weeks after birth (*n* = 2), death within 2 weeks after birth (*n* = 13), and discharge against medical advice within 2 weeks after birth (*n* = 9).

### Data Acquisition and Parameter Definition

All parameters were obtained from the electronic data management systems and the paper file records. The clinical characters of the infants, including gestational age, birth weight, sex, antenatal steroid application, maternal hypertension during pregnancy, maternal gestational diabetes, premature rupture of membranes, type of delivery, fertility treatment, 1st and 5th min Apgar, surfactant, invasive mechanical ventilation, duration of invasive mechanical ventilation, duration of non-invasive mechanical ventilation, duration of oxygen and respiratory support, time when enteral feeding started, time when full enteral feeding achieved (defined as time when parenteral nutrition was ceased), hemodynamically significant patent ductus arteriosus (hsPDA), early of sepsis and late of sepsis (clinical sepsis), and BPD (defined as supplemental oxygen support at 36 weeks post-menstrual age. Preterm infants with early death between day 14 and 36 weeks post-menstrual age, owing to persistent parenchymal lung disease and respiratory failure that cannot be attributable to other neonatal morbidities, would be diagnosed as BPD).

### Nutrition Management

The nutrition protocol for preterm newborns used in this NICU follows the recommendations of ESPHGAN (11–16). A total fluid volume of 80–100 ml/kg/d was started on day 1, with a daily increment of 10–20 ml/kg/d until 150 ml/kg/d. Depending on clinical indications, some infants may have received more or less than 150 ml/kg/d. The initial parenteral solution, including 10% dextrose, amino acid, and calcium, is started soon after birth. A 2 g/kg/day of protein was initiated and increased to 3.5 g/kg/day. Carbohydrates were started with 5–6 g/kg/day and increased to 12–16 g/kg/day, and lipids were started with 1 g/kg/day within 12 h of life and increased to 3.5–4 g/kg/day on the fourth day of life. Serum glucose, urea nitrogen, creatinine and triglyceride levels are monitored three times per week by a bedside chemistry analyzer (Roche, Cobas c111) during the first 2 weeks of life. Enteral diet, mainly own mother's milk, is initiated early within 12 h after birth. Enteral trophic feeding was started at a volume of 10 ml/kg/day (17). The duration of trophic feeding is 7 days for neonates with birth weight <800 g and 5 days for neonates with birth weight between 800 and 1,000 g and 3 days for neonates with birth weight between 1,000 and 1,250 g. Fortification of breastmilk (Human Milk Fortifier, FM85 Nestle, or Similac Abbott) is introduced after 2 weeks of life if a human milk feeding volume of 80–100 ml/kg/d is achieved. If human milk was unavailable or inadequate, the preterm formula was provided. The energy content attributed for each gram of nutrients was carbohydrate-4 kcal, protein-4 kcal, and lipid-9 kcal. The daily nutrition calculation is based on the amount of macronutrients and fluid volume received divided by current weight, considering total parenteral nutrition (TPN), venous fluid volume, and enteral diet. Human milk was calculated as 67 kcal/100 ml for energy, 7.3 g/100 ml for carbohydrates, 1.6 g/100 ml for protein and 3.5 g/100 ml for fats for unfortified breastmilk. This estimate was based on reference nutritional data from previous studies (5, 18). Macronutrients information for formula milk was obtained from the supplied product information sheet.

### Statistical Analysis

As appropriate, data were presented as mean ± standard deviation or median (interquartile range) for all continuous variables. Fisher's exact and chi-square tests were performed for comparing categorical data accordingly. Multivariate logistic regression was used to identify independent variates associated with BPD. All the analysis was performed by IBM SPSS Statistics 24.0. Statistical significance was accepted at *P* < 0.05.

## Results

Overall, *n* = 134 preterm infants were included in the study, of which *n* = 43 (32.1%) developed BPD. The mean gestational age (non-BPD: 27^+3^(26^+5^;28^+4^)weeks vs. with BPD: 26(25;27^+2^)weeks, *p* < 0.001) and mean birth weight (non-BPD: 936 ± 123 g vs. with BPD: 813 ± 120 g, *p* < 0.001) of preterm infants with BPD were significantly lower. Cesarean section rates, fertility treatment, surfactant therapy, duration of invasive mechanical ventilation, duration of non-invasive mechanical ventilation, duration of oxygen and respiratory support, hsPDA were significantly higher in the BPD group (Table 1).

**Table 1:** Patient cohort characteristics separated for bronchopulmonary dysplasia (BPD).

	**Non- BPD *n* = 91**	**BPD *n* = 43**	***P-*value**
Gestational age (weeks)	27^+3^ (26^+5^;28^+4^)	26 (25;27^+2^)	**<0.001**
Birth weight (g)	936 ± 123	813 ± 120	**<0.001**
Male, n (%)	42 (46.2%)	27 (62.8%)	0.072
Cesarean section	49 (53.8%)	14 (32.6%)	**0.021**
Apgar 1 min	8 (5.5;9)	8 (5.5;10)	0.919
Apgar 5 min	10 (9,10)	10 (9.5;10)	0.973
Antenatal steroids	85 (93.4%)	40 (93.0%)	0.934
Fertility treatment	12 (13.2%)	14 (32.6%)	**0.008**
Materal disease-Diabetes	25 (27.5%)	7 (16.3%)	0.156
Materal disease-Hypertension	15 (16.5%)	5 (11.6%)	0.462
Premature rupture of membrane	26 (28.6%)	11 (25.6%)	0.718
Surfactant	44 (48.4%)	33 (76.7%)	**0.002**
Invasive mechanical ventilation	30 (33.0%)	32 (74.4%)	**<0.001**
Duration of invasive mechanical ventilation (days)	0 (0;1)	11 (0.5;23)	**<0.001**
Duration of non-invasive mechanical ventilation (days)	28.5 (13.3;38)	41 (32;60)	**<0.001**
Duration of oxygen and respiratory support (days)	50 (34;63)	84 (73;103)	**<0.001**
Hemodynamically significant patent ductus arteriosus	25 (27.5%)	26 (60.5%)	**<0.001**
Early of sepsis	17 (18.7%)	6 (14.0%)	0.498
Late of sepsis	5 (5.5%)	6 (14.0%)	0.184

*Data are presented as n (%) or median and interquartile range (Q1; Q3). The statistically significant results are indicated by bold print. bronchopulmonary dysplasia (BPD)*.

During the first 2 weeks of life, the average of total caloric intake and the ratio of enteral feeding/ total fluid intake were significantly lower in the BPD group (total caloric intake during the first 2 weeks:91.90 vs. 95.72 kcal/kg/d, *p* = 0.002, the ratio of enteral feeding/total fluid intake during the first 2 weeks: 0.14 vs. 0.18, *p* = 0.004), while the average of total fluid intake, caloric and protein intake from parenteral nutrition did not differ between the groups. The caloric-to-volume ratio of intravenous fluid and ratio of enteral calorie/total calorie intake was significantly higher in the non-BPD group. The human milk proportion in both groups was more than 90% (94.60% in the non-BPD group vs. 97.07% in the BPD group) (Table 2).

**Table 2:** Daily nutritional intake and enteral feeding during the first 14 days of life.

	**Non-BPD *n* = 91**	**BPD *n* = 43**	***P-*value**
Total fluid intake (ml/kg/d)	145.15 ± 4.92	144.24 ± 8.12	0.746
Total caloric intake (kcal/kg/d)	95.72 ± 6.12	91.90 ± 6.88	**0.002**
Total protein intake (g/kg/d)	3.18 ± 0.32	3.10 ± 0.24	0.140
Enteral feeding (ml/kg/d)	25.73 ± 10.11	20.08 ± 11.25	**0.005**
Ratio of enteral feeding/total fluid intake	0.18 ± 0.07	0.14 ± 0.08	**0.004**
Ratio of parenteral calorie / fluid intake from intravenous	0.66 ± 0.05	0.63 ± 0.05	**0.018**
Ratio of parenteral calorie/total calorie intake	0.82 ± 0.07	0.86 ± 0.08	**0.009**
Ratio of enteral calorie/total calorie intake	0.18 ± 0.07	0.14 ± 0.08	**0.009**
Ratio of parenteral calorie /total fluid intake	0.54 ± 0.05	0.54 ± 0.06	0.662
Caloric intake (kcal/kg/d) from parenteral nutrition	78.13 ± 6.81	78.24 ± 7.27	0.933
Protein intake (g/kg/d) from parenteral nutrition	2.77 ± 0.36	2.78 ± 0.28	0.949
Human milk proportion	94.6% (87.2%, 97.4%)	97.1% (90.1%, 100%)	0.05
Time when enteral feeding started (hours)	12 (9, 17)	15 (9, 18)	0.232
Time when full enteral feeding achieved (days)	31 (25;40)	38 (28;49)	0.050

*BPD, bronchopulmonary dysplasia. The statistically significant results are indicated by bold print*.

During the first week of life, the average of total caloric intake, the caloric-to-volume ratio of intravenous fluid and caloric intake from parenteral nutrition were significantly lower in the BPD group (Table 3). During the second week of life, the average of total caloric intake, the ratio of enteral feeding/ total fluid intake and the caloric-to-volume ratio of intravenous fluid were significantly lower in the BPD group (Table 3).

**Table 3:** Daily nutritional intake and enteral feeding during the first week and the second week.

	**Non-BPD *n* = 91**	**BPD *n* = 43**	***P-*value**
**Total fluid intake (ml/kg/d)**			
Week 1	140.19 ± 7.69	139.77 ± 11.28	0.947
Week 2	150.11 ± 5.01	149.17 ± 6.47	0.245
**Total caloric intake (kcal/kg/d)**			
Week 1	87.57 ± 6.70	83.27 ± 7.42	**0.001**
Week 2	104.23 ± 6.69	100.55 ± 8.28	**0.007**
**Total protein intake (g/kg/d)**			
Week 1	3.04 ± 0.22	2.95 ± 0.29	0.055
Week 2	3.32 ± 0.55	3.25 ± 0.26	0.413
**Enteral feeding (ml/kg/d)**			
Week 1	9.55 ± 4.05	8.57 ± 4.15	0.201
Week 2	41.54 ± 17.14	31.95 ± 19.49	**0.005**
**Ratio of enteral feeding/total fluid intake**			
Week 1	0.07 ± 0.03	0.06 ± 0.03	0.177
Week 2	0.28 ± 0.11	0.21 ± 0.13	**0.005**
**Ratio of parenteral calorie/fluid intake from intravenous**			
Week 1	0.62 ± 0.06	0.59 ± 0.06	**0.012**
Week 2	0.70 ± 0.06	0.68 ± 0.07	**0.032**
**Caloric intake (kcal/kg/d) from parenteral nutrition**			
Week 1	80.71 ± 5.93	77.51 ± 7.44	**0.017**
Week 2	75.88 ± 10.69	78.77 ± 11.18	0.157
**Protein intake (g/kg/d) from parenteral nutrition**			
Week 1	2.89 ± 0.22	2.81 ± 0.28	0.089
Week 2	2.65 ± 0.64	2.74 ± 0.42	0.378

*BPD, bronchopulmonary dysplasia. The statistically significant results are indicated by bold print*.

From the univariate analyses, we have identified fifteen significantly different variables between the two groups for the multivariable model: gestational age, birth weight, delivery of cesarean section, fertility treatment, surfactant therapy, duration of invasive mechanical ventilation, hsPDA, ratio of parenteral calorie/total calorie intake, ratio of enteral calorie/total calorie intake, caloric intake during the first week, caloric intake during the second week, ratio of enteral feeding/total fluid intake during the second week, ratio of parenteral caloric intake/fluid intake from intravenous during the first week, ratio of parenteral calorie /fluid intake from intravenous during the second week, caloric intake from parenteral nutrition during the first week. In the multivariable logistic regression model, aparting from the well-known confounders like gestational age, birth weight and invasive mechanical ventilation duration, the ratio of enteral feeding/total fluid intake during the second week was also found to be independently associated with BPD, with an 10% increase in the ratio reducing the risk of BPD by 55.6% (OR 0.444, 95% CI: 0.270–0.731) (Table 4).

**Table 4:** Multivariate logistic regression on factors independently predicting the incidence of BPD.

**Variables**	**β**	**S.E**.	**Wald**	***P***	**OR(95% CI)**
Increase ratio of enteral feeding/total fluid intake during the second week of life by 10%	−0.881	0.254	10.179	0.001	0.444 (0.270–0.731)
Gestational age	−0.659	0.244	7.260	0.007	0.517 (0.320–0.836)
Birth weight	−0.008	0.002	11.079	0.001	0.992 (0.987–0.997)
Duration of invasive mechanical ventilation	0.078	0.030	6.517	0.011	1.081 (1.018–1.147)

*BPD, bronchopulmonary dysplasia*.

*Adjusted for gestational age, birth weight, delivery of cesarean section, fertility treatment, surfactant therapy, duration of invasive mechanical ventilation, hemodynamically significant patent ductus arteriosus, ratio of parenteral calorie/total calorie intake, ratio of enteral calorie/total calorie intake, caloric intake during the first week, caloric intake during the second week, ratio of enteral feeding/total fluid intake during the second week, ratio of parenteral calorie /fluid intake from intravenous during the first week, ratio of parenteral calorie /fluid intake from intravenous during the second week, caloric intake from parenteral nutrition during the first week*.

## Discussion

We have found that a higher ratio of enteral feeding/ total fluid intake during the second week of life was independently associated with a lower risk of BPD in extremely preterm infants (*p* = 0.001). A 10% increase in enteral feeding/total fluid intake ratio significantly reduced the risk of BPD by 55.6% [OR 0.444, 95% CI: 0.270–0.731]. These findings highlight the importance of early enteral nutrition in extremely preterm infants.

Nutrition is essential in the prenatal formation and growth of the lung and influences lung development during postnatal life, especially in early infancy (19). Aggressive nutritional regimes of early parenteral and enteral dietary support are associated with improved growth and less neonatal morbidities (20). Since the defect of enteral caloric intake in most cases is compensated for parenteral nutrient supply, the importance of enteral feeding could not be replaced by parenteral nutrition. Previous studies have focused on the benefits of early enteral feeding to reduce NEC and late-onset sepsis but less on the benefits for BPD. In the current study, a higher ratio of enteral feeding/total fluid intake during the second week of life was significantly associated with a lower risk of BPD. This finding is consistent with the other two studies (18, 21), although the population in our study was younger and smaller. However, Thiess (6) reported that enteral nutrition supply tended to be higher in the no/mild BPD group but without statistical significance after risk adjustment. As the period of their study lasted 10 years, the improvement in neonatal care, especially the ventilation strategy, might mask the effect of nutrition. Uberos (19) and Wemhöner (22) reported that enteral feeding was significantly lower in BPD infants. Still, they didn't provide a correlation coefficient between enteral feeding and BPD.

In this study, we have found a 10% increase in enteral feeding/total fluid intake ratio during the second week significantly reduced BPD risk by 55.6%. The ratio of enteral feeding/ total fluid intake during the first week did not differ between the groups mainly due to trophic feeding. Apart from the adequate nutrition needed for postnatal lung development, the beneficial effect of early aggressive enteral nutrition could be the protective effect by gut microbiota and metabolites associated with increased enteral feeding (23) and easier regulation on enteral fluid intake in those infants, compared with direct intravenous input. Other protective mechanisms involved might be the high amount of active proteins in early breast milk, such as whey proteins, lactoferrin, other antioxidant components, and immune activity factors. However, the human milk proportion was similar between BPD infants (97.07%) and non-BPD ones (94.60%), limiting the differentiated effect caused by breast milk in this study.

Similar to the finding in our study, many have reported a lower energy intake in early life in BPD infants (4, 5, 9, 19, 20), although nutrition strategies vary among different NICUs. The undernutrition in BPD infants is multifactorial. Feeding intolerance is common in extremely premature infants, possibly resulting from insufficient gut perfusion, triggered by prematurity-associated morbidities like respiratory instability and hemodynamically significant PDA. Furthermore, the subjective assessment of feeding tolerance might also hinder the increase in feeding volume (6). We found that the caloric-to-volume ratio of intravenous fluid was significantly lower in the BPD group. This may be secondary to intravenous administration of calorie-deficient fluid, such as inotropic drug infusion and blood product transfusion, more often used among BPD infants. Therefore, parenteral nutrition should be more precisely monitored in those infants. To be more specific, the caloric-to-volume ratio in the parenteral nutrition fluid should be increased to compensate for the nutritional deficiencies caused by low enteral feeding and increased demand for calorie-deficient fluid intake.

We acknowledge several limitations in the current study. Apart from the retrospective nature, the energy calculation of breast milk was uniform since we did not analyze the nutrient contents of breast milk. Furthermore, we did not validate the causality between enteral feeding and BPD in an external cohort. An intervention study is needed to confirm the beneficial effect of aggressive enteral feeding in early life on BPD in extremely preterm infants.

## Conclusion

Our study showed that a higher enteral feeding/ total fluid intake ratio was associated with a lower risk for BPD in extremely preterm infants. Early and rapidly progressive enteral nutrition should be encouraged in these infants.

## Data Availability Statement

The raw data supporting the conclusions of this article will be made available by the authors, without undue reservation.

## Ethics Statement

The studies involving human participants were reviewed and approved by the Shenzhen Maternity and Child Healthcare Hospital Institutional Ethical Committee. Written informed consent from the participants' legal guardian/next of kin was not required to participate in this study in accordance with the national legislation and the institutional requirements.

## Author Contributions

BL and XC designed the research subject. XX and XL collected and analyzed the data. JZ and ZH realized the statistical tests. BL wrote the manuscript. XC critically revised the manuscript. All authors read and approved the final manuscript.

## Funding

This study was supported by Shenzhen Science and Technology Innovation Committee (JCYJ20190809170009528, XC), Shenzhen Fund for Guangdong Provincial High level Clinical Key Specialties (SZGSP009), and Guangdong Basic and Applied Science Committee (2021A1515011194, XC).

## Conflict of Interest

The authors declare that the research was conducted in the absence of any commercial or financial relationships that could be construed as a potential conflict of interest.

## Publisher's Note

All claims expressed in this article are solely those of the authors and do not necessarily represent those of their affiliated organizations, or those of the publisher, the editors and the reviewers. Any product that may be evaluated in this article, or claim that may be made by its manufacturer, is not guaranteed or endorsed by the publisher.
